# Ammonium nanochelators in conjunction with arginine-specific enzymes in amperometric biosensors for arginine assay

**DOI:** 10.1007/s00604-023-06114-1

**Published:** 2023-12-22

**Authors:** Nataliya Stasyuk, Galina Gayda, Wojciech Nogala, Marcin Holdynski, Olha Demkiv, Lyubov Fayura, Andriy Sibirny, Mykhailo Gonchar

**Affiliations:** 1 Institute of Cell Biology, National Academy of Sciences of Ukraine, Lviv, 79005 Ukraine; 2 Institute of Physical Chemistry, Polish Academy of Sciences, Kasprzaka 44/52, 01-224 Warsaw, Poland; 3 Department of Biotechnology and Microbiology, Rzeszow University, 35-601, Rzeszow, Poland

**Keywords:** Amperometric biosensor, L-arginine, L-arginine oxidase, Arginine deiminase, Arginase, Urease, Ammonium sensing, Nanoparticles

## Abstract

Amino acid L-arginine (Arg), usually presented in food products and biological liquids, can serve both as a useful indicator of food quality and an important biomarker in medicine. The biosensors based on Arg-selective enzymes are the most promising devices for Arg assay. In this research, three types of amperometric biosensors have been fabricated. They exploit arginine oxidase (ArgO), recombinant arginase I (ARG)/urease, and arginine deiminase (ADI) coupled with the ammonium-chelating redox-active nanoparticles. Cadmium-copper nanoparticles (nCdCu) as the most effective nanochelators were used for the development of ammonium chemosensors and enzyme-coupled Arg biosensors. The fabricated enzyme/nCdCu-containing bioelectrodes show wide linear ranges (up to 200 µM), satisfactory storage stabilities (14 days), and high sensitivities (A⋅M^−1^⋅m^−2^) to Arg: 1650, 1700, and 4500 for ADI-, ArgO- and ARG/urease-based sensors, respectively. All biosensors have been exploited to estimate Arg content in commercial juices. The obtained data correlate well with the values obtained by the reference method. A hypothetic scheme for mechanism of action of ammonium nanochelators in electron transfer reaction on the arginine-sensing electrodes has been proposed.

**Graphical abstract:**

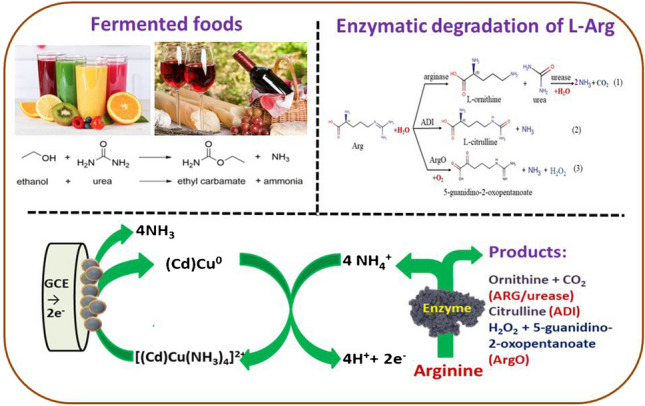

**Supplementary Information:**

The online version contains supplementary material available at 10.1007/s00604-023-06114-1.

## Introduction

Since L-arginine (Arg) is a common component of food and biological fluids, it can be used as an indicator of food quality [1–4] and an important biomarker in clinical diagnosis [5–11]. Thus, to monitor Arg in the food industry and in medicine, simple, rapid, and selective approaches are needed.

Numerous optical and electrochemical analytical methods for Arg determination have been developed to date [2, 12–15]. Simple and quick optical methods are based on the well-known Sakaguchi reaction, which involves the formation of color product between Arg and 8-hydroxyquinoline [12, 16]. However, these methods possess certain disadvantages, such as low specificity and insufficient selectivity. State-of-the-art methods for determining Arg, which rely on the use of advanced equipment, show promise for use in clinical and industrial laboratories [2, 13, 14, 17–19]. However, these methods suffer from limited selectivity, are laborous and require highly trained personnel. These disadvantages can be overcome by enzymatic approaches [20–22].

The enzymes metabolizing Arg exhibit high selectivity towards their natural substrate, so they are ideal candidates for biorecognition elements in optical and electrochemical biosensors of Arg [23–26]. Arginase I (ARG, EC 3.5.3.1, L-arginine amidino hydrolase) is an enzyme expressed in the human liver and critical to the urea cycle. It promotes the hydrolysis of Arg to L-ornithine and urea (Eq. 1). Arginine deiminase (ADI, EC3.5.3.6, L-arginine amino hydrolase) catalyzes the deamination of arginine to yield ammonia as the by-product (Eq. 2). ArgO (EC 1.4.3.25, L-arginine oxygen oxidoreductase) catalyzes the oxidation of Arg to form ammonia and hydrogen peroxide (Eq. 3) as the by-products. For enzymatic detection of Arg, it was suggested to combine the cascade reactions based on the application of ARG, urease, and glutamate dehydrogenase and measured oxidized NADH at 340 nm [20].

As an alternative to enzymatic approaches, chemo-sensors based on the use of electrocatalytic nanomaterials have been proposed. However, although they are sensitive to Arg, the non-enzymatic sensors exhibit low selectivity [19, 27–31].

Thus, all known approaches for Arg determination require considerable time, skillful lab personnel, advanced equipment, the need for cascades of enzymes, and expensive reagents [2, 13, 18, 32]. Furthermore, since they do not provide the possibility of real-time analysis, the development of electrochemical biosensors (BSs) for Arg monitoring remains of primary interest.
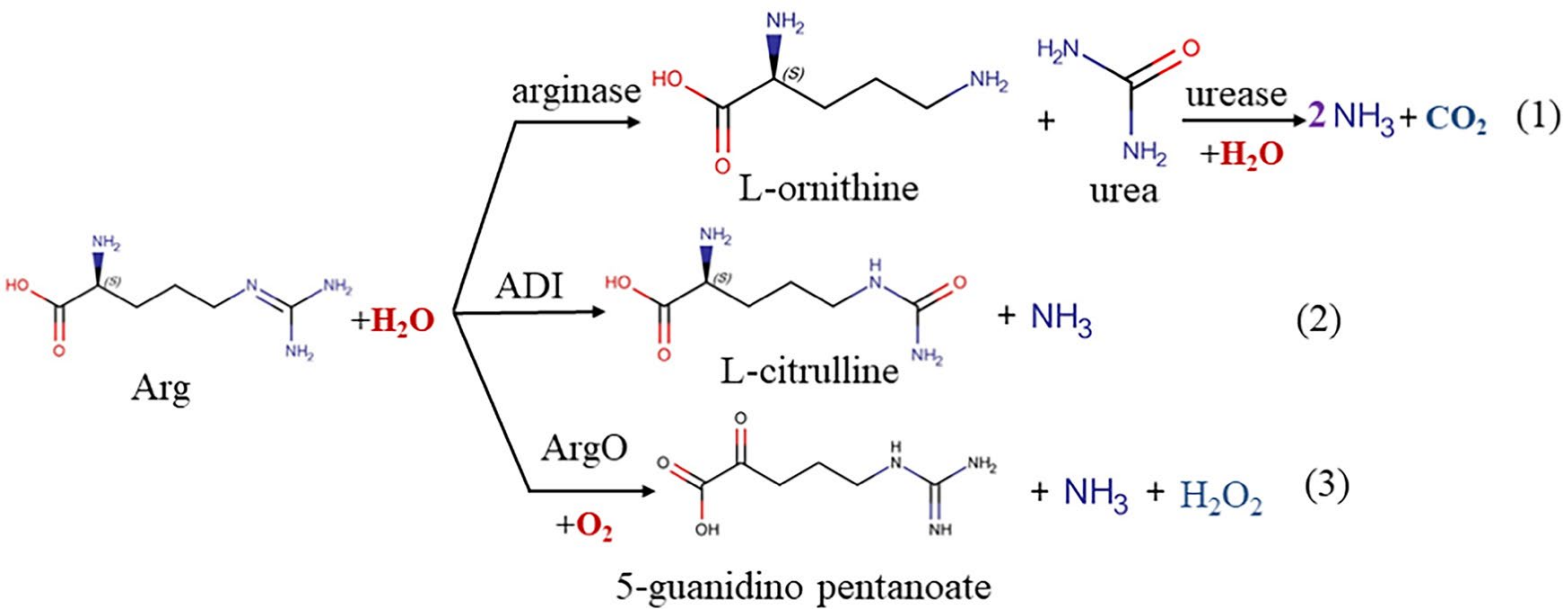


To estimate the level of Arg, the electrochemical BSs which detect the ammonium ions produced during the enzymatic digestion of Arg have been suggested and described in detail in the reviews [23–25] and our previous reports [33–35]. Numerous designs of potentiometric and conductometric BSs based on ammonium-selective electrodes have also been reported [23–25, 32, 36].

Despite the popularity of potentiometric BSs, they have a number of disadvantages, namely, the dependence of membrane potential on presence of other ions, temperature, ionic strength of the solution, prolonged response time, and short-term operational stability due to the quick destruction of the polymeric membrane [25]. Amperometric BSs (ABSs) are more promising for ammonia monitoring due to their higher sensitivity and selectivity as well as easy integration into continuous analytic systems [23–26]. Several ABSs for Arg assay have been developed and described [23–26, 37–39]. Some of them are based on the application of Arg-selective hydrolases, in particular, arginase/urease or ADI co-immobilized with conductive polymers in the bioselective layer [21, 26, 32–35]. Other ABSs are based on a combination of ArgO with natural or artificial peroxidase [40–42]. However, their effectiveness as sensors in real applications is limited by several drawbacks, including insufficient operational stability [25, 39]. 

ADI and ArgO are the best candidates for the construction of mono-enzyme ABSs sensitive and selective towards Arg. Additionally, the combination of natural enzymes with appropriate nanomaterials seems to be the most optimal approach to develop advanced sensor devices with improved characteristics, namely, high stability, sensitivity, and selectivity to the target analyte [13–15, 25, 26, 34]. To date, there are many reports on ArgO-containing ABSs which rely on the principle of detection of hydrogen peroxide as a by-product of Arg degradation. However, there are no reports on ArgO-based ABSs which detect ammonium, so far.

Nowadays, limited data are available on the mono-enzymatic ABSs which are based on ammonia/ammonium-sensitive nanocomposites—“nanochelators”—able to form redox-active coordination compounds with ammonia [43]. The crystal structure of more than twenty coordination compounds formed by Cu^2+^ ions with ammonia has been elucidated [44]. Some of these complexes are electroactive and could be detected by amperometry. Recently, metal oxides in the form of NPs or nanocomposites such as WO_3_ [45], Cu/Zn(Hg)S [34], In_2_O_3_ [46], Au/PANi [47], and Ni/graphene/PANi [48] have been reported. They are useful for the amperometric detection of ammonia. Only a few papers focus on the ABSs on Arg, which are based on metal oxides and sulfur-containing NPs as ammonia chemosensors [34, 49]. The construction of bioelectrodes based on the enzymes conjugated with metallic ammonia-sensitive NPs in a bioselective layer remains a significant challenge. In view of this, the Arg-selective enzymes ARG, ADI, and ArgO appear to be promising tools for the development of ABSs on Arg.

The aim of the current research is to design Arg selective ABSs using metal hybrid NPs as redox-active ammonia nanochelators. The research focuses on the synthesis and characterization of nanocomposites and their application to biosensors’ development. We also report the application of the constructed enzyme-based ABSs to the analysis of Arg. The proposed bioelectrodes are based on the combination of metal hybrid NPs and Arg-selective enzymes (ARG, ADI and ArgO). The hypothetic scheme for mechanism of action of ammonium nanochelators in electron transfer reaction on the arginine-sensing electrodes has been proposed.

## Materials and methods

### Chemicals

Urease (EC 3.5.1.5, type IX from Jack Beans, 26,100 U g^−1^)**,** L-arginine (Arg), Cetyl trimethyl ammonium bromide (CTAB, 99%), Copper(II) sulphate (CuSO_4_, 99.5%), Cadmium(II) chloride (CdCl_2_, 99.5%), sodium borohydride (NaBH_4_, 99%), glutaraldehyde (25%), and cysteamine. Amino acids were obtained from Sigma-Aldrich (Germany).

### Synthesis and characterization of metallic nanocomposites

The copper NPs (nCu) were obtained by chemical reduction using sodium borohydride as a reducing agent. The Cd(core)/Cu (shell) NPs (nCdCu) were synthesized by the chemical bath deposition method. Firstly, 0.5 mL 0.05 M CdCl_2_ was injected into the 15 mL 10 mM CTAB solution and stirred for 5 min. Then, 1 mL 0.05 M Na_2_S was added to the mixture, vigorously stirred, and heated at 100 °C for 10 min. The synthesized yellow NPs of CdS (nCdS) were used as seeds for the following stage. A 5 mL nCdS solution was injected into the growth mixture containing 10 mL 0.05 M CuSO_4_ with the following addition of 3 mL 0.05 M Na_2_S and heating at 100 °C for 10 min. The synthesized nCdCu are of a dark brown color. All obtained NPs were isolated from the reaction mixture by centrifugation (8000 g, 30 min) and washed with 5 mM phosphate buffer, pH 7.0 and water. The final nCdCu and nCu precipitates were dried (at 100 °C for 24 h).

The NPs ZnCu, CuCeAu, and CuCe were synthesized by the chemical deposition method as described earlier [50]. The size analysis of NPs was evaluated by the use of FEI Nova NanoSEM 450 and SEM-microanalyzer REMMA-102–02.

The surface chemical composition measurements were performed by the X-ray photoelectron spectroscopy (XPS) using a Microlab 350 (Thermo Electron, East Grinstead, UK) spectrometer. Survey spectra were collected using the X-ray excitation source (AlKα anode: power 300 W, voltage 15 kV, beam current 20 mA) with pass energy at 100 eV and energy step at 1 eV. The high-resolution XPS spectra were recorded separately, using smaller energy step (0.1 eV) and lower pass energy 40 eV. All the collected XPS data were fitted using an asymmetric Gaussian/Lorentzian mixed function. The measured binding energies were corrected in reference to the energy of C 1 s at 284.8 eV. CasaXPS software was used to process the data [51].

### Enzymes preparations

Human liver ARG I was isolated from the recombinant yeast *Ogataea polymorpha pGAP1–HsARG1* (*leu2car1 Sc:LEU2*) and purified by affinity chromatography (up to specific activity 150 U⋅mg^−1^ of protein) as it was described earlier [22] and kept in 50 mM Tris–HCl buffer, pH 8.8 (TB) at + 4 ^∘^C till usage.

Bacterial *Mycoplasma hominis* ADI was isolated from the recombinant cells of *Escherichia coli* BL21(DE3)/pET3d-ADI, purified by two-step column chromatography (up to 180 U⋅mg^−1^) [22] and kept in 50 mM phosphate buffer, pH 7.0 (PB) at + 4 °C until usage.

ArgO from mushroom *Amanita phalloides* was purified by ion exchange chromatography on Toyopearl DEAE-650 M (up to 0.56 U⋅mg^−1^) [41] and stored in 70% ammonium sulphate/PB at + 4 °C.

### Development of the nanostructured bioelectrodes

All electrochemical measurements were conducted using a Metrohm Autolab PGSTAT30 with Ag/AgCI/3 M KCI and platinum (Pt) wire as reference and counter electrodes, respectively. A glassy carbon rod (GCE, Mineral, Poland) with a diameter of 3.05 mm (surface area 7.06 mm^2^) was used as a working electrode. Before biosensor preparation, the electrode was polished in 20% isopropanol water solution in the ultrasonic cleaning bath.

To obtain the electrode modified with NPs, a 2 µL suspension of NPs (1 mg·mL^−1^) was dropped onto the GCE and air-dried. Then, an aliquot of mixture containing cysteamine (1 mM) and glutaraldehyde (1%) was placed onto the surface of GCE/NPs and air-dried. Finally, the activated NPs/GCE was rinsed with PB and used for enzyme immobilization by the cross-linking method.

Electrophoretically homogeneous Arg-selective enzymes were applied to the construction of ABSs. To fabricate the ADI or ArgO-based bioelectrodes, 2 µl of the correspondent enzyme solution was dropped on the surface of the activated NPs/GCE. To construct the ARG-based bioelectrode, 2 µl aliquots of urease and ARG were consecutively dropped on the activated NPs/GCE and air-dried at r.t. All constructed bioelectrodes were stored at + 4 °C in the vapors of the correspondent buffers (PB or TB) until usage.

The obtained NPs were screened for their redox activity in increased ammonium concentrations using cyclic voltammetry (CV) *vs.* Ag/AgCI/3 M KCI as the reference electrode. The highest signal at – 150 mV was chosen as 100%.

### Arg assay in fruit juices

The fabricated amperometric biosensors were checked on the model of three commercial juices “Tymbark” (LtD Tymbark, Poland): apple, raspberry, and orange ones. Arg concentrations in all samples were measured using a standard addition test (SAT). The reference Arg assay was performed using the enzymatic analytical kit “Argitest” [22].

### Statistical analysis

All experiments were carried out three times (*n* = 3) and measurements were performed in two parallels. The statistical parameters and all figures were calculated and built using Origin 8.5 Pro.

Sensitivity (A‧M^−1^‧m^−2^) was calculated as follows: Sensitivity = B/S, where B is the slope for the dependence of current on analyte concentration in linear range (A‧M^−1^) and S is the surface area of the working electrode (m^2^).

The limit of detection (LOD) was calculated by using the standard deviation (SD) of the blank current signals and the B value according to the formula: LOD = (3 ∗ SD/B).

## Results and discussion

### Selection of the most chemically active ammonium-sensitive nanocomposites and their characterization

The purpose of the current research was to develop novel ABSs for Arg analysis which are based on Arg-selective enzymes coupled with new ammonium-sensitive NPs as redox-active nanochelators and to demonstrate the applicability of the resulting ABSs for Arg determination in food samples.

To date, there is limited data on mono-enzymatic ABS based on Arg-selective enzymes that control ammonium as a product of the enzymatic cleavage of Arg. All of the reported ABSs are based on potentiometric detectors or a combination of the cascade of enzymes. Recently, numerous attempts have been made to search the prospective materials, for ammonium assay have been made. Among them, some metal oxides and sulfides are promising [45, 46, 49]. In the current paper, a number of NPs were synthesized using chemical reduction and bath deposition methods (see Section “Synthesis and characterization of metallic nanocomposites”). The obtained NPs were screened on their redox activity in the presence of ammonium using cyclic voltammetry (CV). Since nCdCu and nCu were shown to display the highest activity (see Supplementary Information, Fig. S1), these NPs were utilized for further studies.

Amperometric characteristics of the most electroactive nCdCu and nCu are provided in Figure S2. CV profiles for both types of electrodes showed a strong peak caused by the reduction of the formed ammonium-NPs complex occurring at potentials between 0 mV and – 350 mV. For the amperometry analysis, the potentials of – 150 mV and – 300 mV were chosen as the optimal ones for nCdCu- and nCu-modified electrodes, respectively (Fig. S2). The maximum current responses of the nCdCu/GCE and nCu/GCE to injected ammonium chloride (as a source of NH_4_^+^ ions) were 22,411 ± 60 nA and 12,800 ± 40 nA with the values of K_M_^app^ 1.6 ± 0.1 mM and 2.15 ± 0.15 mM, respectively. Thus, the synthesized nCdCu and nCu are effective redox-active nanochelators for the ammonium and prospective platforms to develop ABSs on Arg.

**Fig. 1:**
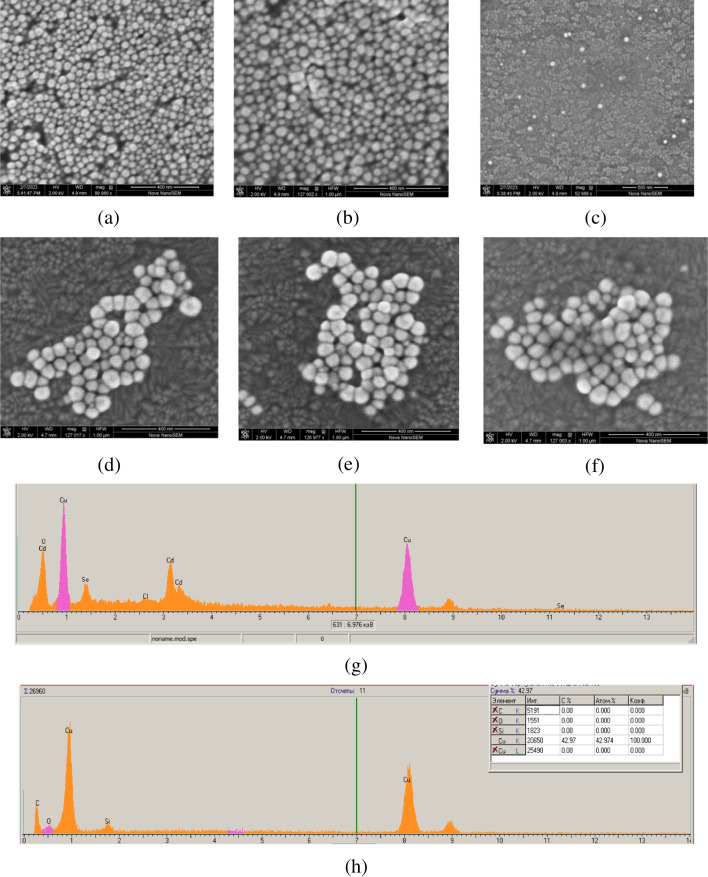

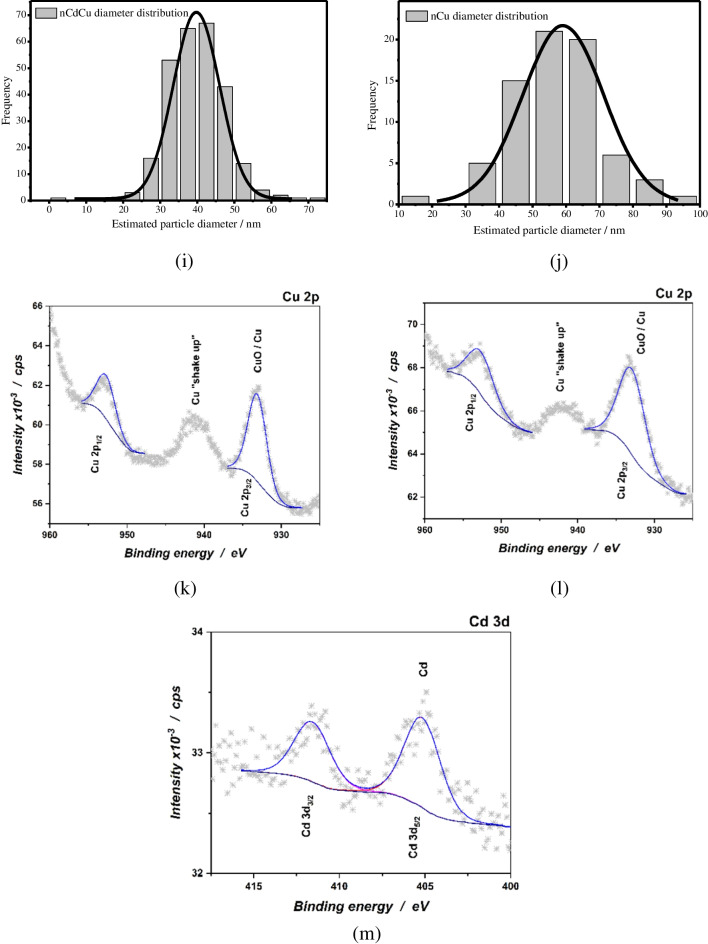
Morphological properties of NPs: SEM images (**a–f**); X-ray spectral microanalysis (**g, h**), the Gaussian distribution by sizes (**i, g**), and XPS spectra of Cu 2p and Cd 3d (**k–m**) for nCdCu (**a–c, g, i, k–l**) and nCu (**d–f, h, j, m**), respectively

To investigate the morphology of the synthesized NPs (nCdCu and nCu) as the best sensing materials for ammonium ions, SEM in combination with EDS analysis was performed (Fig. 1). Since nCdCu and nCu have the shape of spheres, the Gaussian distribution demonstrates the average sizes of nCdCu and nCu to be 39.5 nm and 57 nm, respectively. Figure 1g–h displays the XRM spectrum of the nCdCu and nCu which mainly presents the peaks of Cd, Cu on the Si surface.

X-ray photoelectron spectroscopy was used for chemical characterization of the obtained nanoparticles. The analysis of the survey spectra revealed presence of the same elements previously observed using EDS technique. Figure 1 shows Cu 2p high-resolution spectra for nCu and nCdCu samples. For the tested NPs, it can be seen that the position of the Cu 2p_3/2_ peak corresponds to an energy of ~ 933.0 eV, which suggests the presence of the metallic copper Cu^0^ or mixture of metallic state and non-stoichiometric oxidized form of Cu^+^ [52]. Moreover, Cd 3d core level spectra were recorded for nCdCu sample. Peak maximum of the deconvoluted peak of Cd 3d5/2 can be attributed to metallic state of Cd^0^, with binding energy at ~ 405.3 eV [52].

### Electrochemical properties of NPs/GCE modified with Arg-selective enzymes

**Fig. 2:**
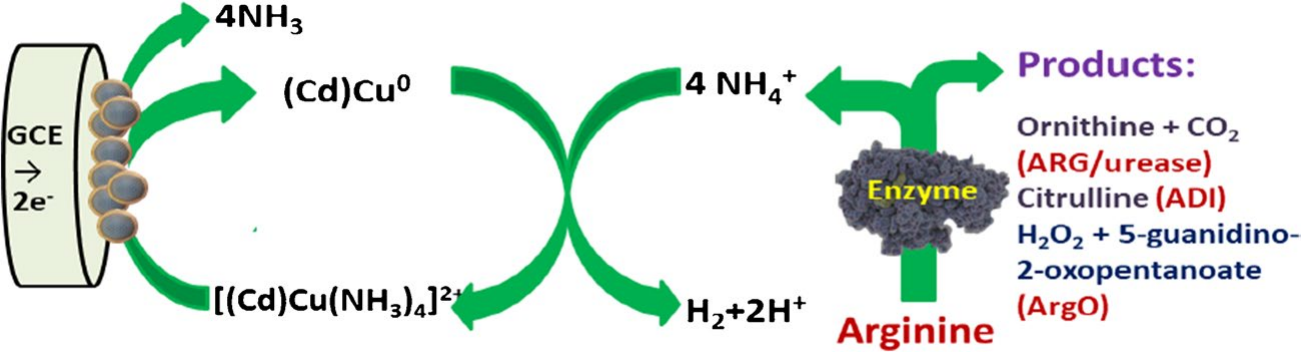
The hypothetical mechanism for Arg detection by the developed ABSs

To form an enzymatic layer, the enzymes (ArgO, ADI or ARG in combination with urease) were covalently conjugated to the surface of NPs/GCE (see Section “Arg assay in fruit juices”). The assay of Arg by the designed bioelectrode is based on monitoring of ammonium ions (NH_4_^+^) generated in an enzymatic layer and finally sensed by the NPs/GCE electrode (see Fig. 2). The first stage of biorecognition is the ARG/urease, ADI, or ArgO catalyzed Arg conversion to correspondent by-product (see Eqs. 1–3) and ammonium ions. The resulting NH_4_^+^ ions diffuse further to the NPs (nCu or nCdCu) layer and trigger the formation of the electroactive complex with the structure [Cu(NH_3_)_4_]^2+^. It is well known from the literature that Cu(I) and Cu(II) can exist as their ammine complexes in aqueous solutions containing dissolved ammonia [53]. In aqueous media, the cathodic reduction of copper(II) ammines proceeds stepwise to Cu(0) through Cu(I) ammines. The electroactivity of the produced complex could be explained by the ability of the bonded Cu^2+^ ions to participate in the electron transfer reaction. The electrochemical transformation of the produced complexes NH_4_^+^ with NPs (Cd(core)/Cu(shell) or nCu) on the GCE surface can possibly occur with the following stages (Eq. 4 – 9):

1. Nanoparticle surface oxidation by ammonium cations and complexation:
  4 $$\mathrm{CdCu}+4\;\mathrm{NH}_4^+\rightarrow\lbrack\text{CdCu}{{({\text{NH}}_3)}_4\rbrack}^{2+}+{\text{H}}_2+{2\;\mathrm H}^+$$
  5 $$\mathrm{Cu}+4\;\mathrm{NH}_4^+\rightarrow\lbrack\text{Cu}{{({\text{NH}}_3)}_4\rbrack}^{2+}+{\text{H}}_2+{2\;\mathrm H}^+$$
2. Electroreduction and dissociation of the complex. The reduction of the produced complex results in the cathodic current signal, whose value is correlated with the level of Arg in the sample:
  6 $${\lbrack\text{CdCu}{({\text{NH}}_3)}_4\rbrack}^{2+}+\text{e}^-\rightarrow{\lbrack\text{CdCu}{({\text{NH}}_3)}_2\rbrack}^++2\;{\mathrm{NH}}_3$$
  7 $${\lbrack\text{CdCu}{({\text{NH}}_3)}_2\rbrack}^++\text{e}^-\rightarrow\mathrm{CdCu}+2\;{\mathrm{NH}}_3$$
  8 $${\lbrack\text{Cu}{({\text{NH}}_3)}_4\rbrack}^{2+}+\text{e}^-\rightarrow{\lbrack\text{Cu}{({\text{NH}}_3)}_2\rbrack}^++2\;{\mathrm{NH}}_3$$
  9 $${\lbrack\text{Cu}{({\text{NH}}_3)}_2\rbrack}^++\text{e}^-\rightarrow\mathrm{Cu}+2\;{\mathrm{NH}}_3$$

In general, any of the processes (4)–(5) might be rate-determining steps, and these can be distinguished by the nature of the dependence of the reaction rate upon electrode potential. By applying a negative potential to the electrode modified with nCdCu or nCu, reactions (6–7) and (8–9) are fast enough that reactions (4) and (5) limit the rate of the overall process. Then, the overall electrode process rate depends on the concentration of the ammonium ions.

The hypothetical mechanism for Arg detection by created bioelectrodes is based on the ability of metallic NPs to be oxidized by NH_4_^+^ with the formation of surface complex compounds with ammonia, resulting in changing their redox potential. The scheme of possible electron relocation between a bioselective layer and the GCE surface is shown in Fig. 2. The electrocatalytic activity of the nCu or nCdCu layered on GCE was tested by the use of cyclic voltammetry under the consequent injections of increased amounts of ammonia (Fig. S1). The CV for nCdCu (Fig. S1a) demonstrated that a cathodic peak, contributed by ammonium ions, appeared at a potential of – 150 mV. An anodic peak (is due to oxidation of generated H_2_) was detected at ca. + 200 mV. The results of amperometry analysis and calibration graphs for nCdCu/GCE and nCu/GCE at – 150 mV and – 300 mV are presented in Fig. S2a–b, respectively.

#### nCdCu as a chemosensor on ammonium ions

**Fig. 3:**
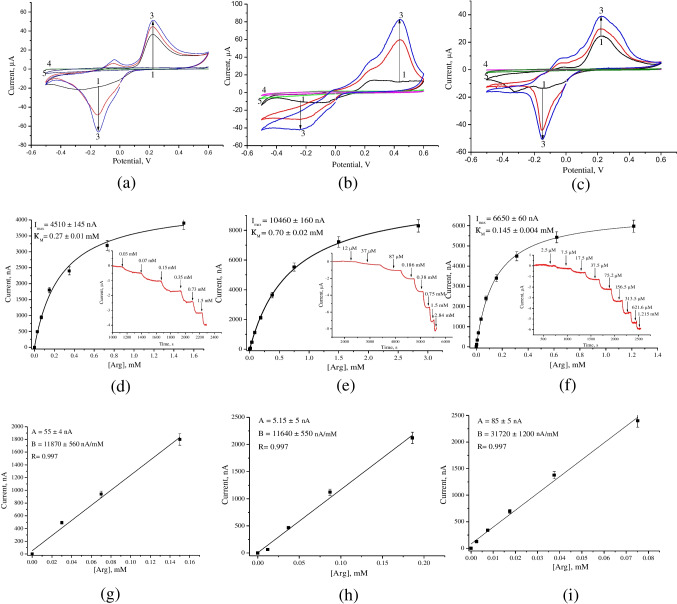
Electrochemical characteristics of GCEs modified with nCdCu and ArgO (**a, d, g**), ADI (**b, e, h**), and ARG/urease (**c, f, i**) in the presence of increased concentrations of Arg. CVs (**a–c**) under injected Arg up to concentrations 0.2 mM (2), 0.5 mM (3) in comparison with nCdCu/GCE (1) and unmodified GCE (4), and enzyme/GCEs (5) (as a control electrodes) in the presence of 0.5 mM Arg; amperometric responses (**d–f**) and calibration curves (**g–i**) of peak current vs total Arg concentration. Conditions: CV scan rate 5 mV·s^−1^ *vs.* Ag/AgCl, pH 7.5 (for ADI- or ArgO-based sensor) or 50 mM TB, pH 8 (for ARG/urease sensor); working potentials for ArgOx/nCdCu/GCE, ARG/urease/nCdCu/GCE, and ADI/nCdCu/GCE are as follows: – 150 mV, – 150 mV, and – 200 mV, respectively

The three types of nCdCu-modified GCEs were studied, namely, ARG in combination with urease (designated as ARG/urease/nCdCu/GCE), ADI with nCdCu (ADI/nCdCu/GCE), and ArgO with nCdCu (ArgOx/nCdCu/GCE). The electrocatalytic activity of three bioelectrodes was evaluated by using CV under the addition of Arg (Fig. 3a–c). Each obtained CV profile demonstrated two peaks under injected Arg which correspond to cathodic and anodic peaks with maximums at – 147 mV and + 221 mV (for the ARG/urease/nCdCu/GCE and ArgOx/nCdCu/GCE) to + 440 mV (for ADI/nCdCu/GCE), respectively. Amperometric investigations were conducted at the selected optimal working potentials for each type of bioelectrode; in particular, for ArgOx/nCdCu/GCE, ARG/urease/nCdCu/GCE, and ADI/nCdCu/GCE, the optimal potentials were chosen as follows: – 150 mV, – 150 mV and – 200 mV, respectively. The results of amperometric studies as well as calibration graphs at optimal working potentials (the highest difference between analyte and background current) are shown in Fig. 3 and Table S1. It should be noted that nCdCu/GCE was also tested as a control electrode and no amperometric signals were observed under injected Arg (Fig. 3a–c, curve 4).

As shown in Table S1, ammonia-selective nCdCu had a significant positive effect on biosensor sensitivity in comparison to electrodes modified with other NPs (Table 1). For example, the biosensor with architecture ARG/urease/nCdCu/GCE was 41-fold more sensitive than the amperometric biosensor based on ARG, urease, and ammonia-sensitive polyaniline film (ARG-urease/PANI/PtE) [35]. The increase in sensitivity of ARG/urease/nCdCu-based biosensor can be explained by higher redox-activity of nCdCu (by 5 times) in comparison with PANi. The sensitivity of ArgO and ADI-based bioelectrodes was also higher than that previously reported; it was similarly determined by the increase of the GCE surface area due to the small-sized nCdCu resulting in a subsequent increase of local enzyme concentrations in the bioselective layer.

**Table 1:** Comparison of the most effective amperometric biosensors for Arg assay

Bioelectrode	Monitored product	Working potential, mV	Sensitivity, A·M^−1^·m^−2^	Linear range, µM	LOD, µM	Reference
ArgO/nCeCu/^1^GE	H_2_O_2_	– 150	1630 ± 92	5–100	^2^N.d	[41]
ArgO/nNiPtPd/GE			578 ± 5	10–250		
ArgO/gCuHCF/GE			602 ± 42	10–100		
ArgO/ Phenol red		–	–	100–1000	16	[54]
ArgO/nCu/^5^GCE	NH_4_^+^	– 300	2300 ± 15	2–18	0.7	Current paper
ArgO/nCdCu/GCE		– 150	1700 ± 80	30–160	5.5	
ADI/PANi/Nafion/Pt-^3^SPE		– 350	684 ± 32	3–200	1.0	[33]
ADI/Cu/Zn(Hg)S/GE		– 200	1570	13–800	4.3	[34]
ARG -urease/PANI/PtE			110 ± 1.3	70–600	38	[35]
^4^p-cells/urease/PANI/PtE			14 ± 1.1	Up to 600	N.d	[55]
ARG -nAu/p-cells/urease /PANi/PtE			357 ± 24	10–700		
ADI/nCu/GCE		– 300	505 ± 3	6 – 130	1.8	Current paper
ARG-urease/nCu/GCE			780 ± 55	6 – 50	1.75	
ARG-urease/ nCdCu/GCE		– 150	4500 ± 170	2–80	0.6	
ADI/nCdCu/GCE		– 200	1650 ± 75	12–200	3.6	

^1^*GE* graphite electrode

^2^*N.d.* not detected

^3^*SPE* screen-printed electrode

^4^*p-cells* permeabilized cells of the yeast *O. polymorpha*

^5^*GCE* glassy carbon electrode

#### nCu as a chemosensor on ammonium ions

**Fig. 4:**
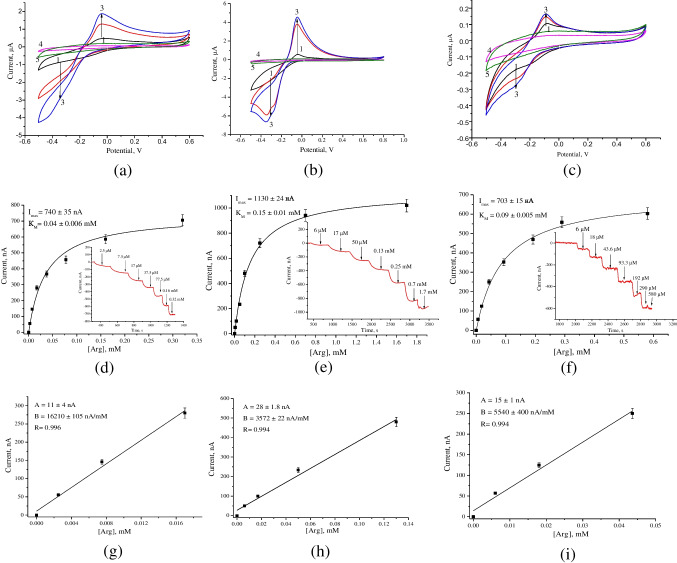
Electrochemical characteristics of GCEs modified with nCu and ArgO (**a, d, g**), ADI (**b, e, h**), and ARG/urease (**c, f, i**) in the presence of increased concentrations of Arg. CVs (**a–c**) under injected Arg up to concentrations 0.2 mM (2), 0.5 mM (3) in comparison with nCdCu/GCE (1) and unmodified GCE (4), and enzyme/GCEs (5) (as a control electrodes) in the presence of 0.5 mM Arg; amperometric responses (**d–f**) and calibration curves (**g–i**) of peak current vs total Arg concentration. Conditions: CV scan rate 5 mV·s^−1^ *vs.* Ag/AgCl, pH 7.5 (for ADI- or ArgO-based sensor) or 50 mM TB, pH 8 (for ARG/urease sensor); working potential for all bioelectrodes was ArgOx/nCdCu/GCE, ARG/urease/nCdCu/GCE and ADI/nCdCu/GCE was – 300 mV

To date, there are no reports regarding the ABSs based on nCu for the detection of ammonia. We constructed ABSs based on enzymes selective to Arg and nCu for monitoring ammonia which is produced during the enzymatic cleavage of Arg. The electrocatalytic activities of the nCu conjugated with the enzymes were tested using CV and amperometry (Fig. 4). The obtained CVs (Fig. 4) demonstrated strong reduction peaks at near – 300 mV which was chosen as an optimal working potential for three types of bioelectrodes. The obtained data of amperometry and correspondent calibration graphs at potential – 300 mV are shown in Fig. 4 and Table 2, respectively.

**Table 2:** Detection of Arg level (mM) in the samples of commercial juices

Method	Biosensor						Reference enzymatic method	
	ADI/nCdCu/GCE		ARG/urease/nCdCu/GCE		ArgO/nCdCu/GCE			
Juice	Arg	CV, %	Arg	CV, %	Arg	CV, %	Arg	CV, %
Apple	2.52 ± 0.12 *p* > 0.05	4.7	2.35 ± 0.10 *p* > 0.05	4.25	2.26 ± 0.15 *p* > 0.05	6.6	2.46 ± 0.07	2.8
Raspberry	2.15 ± 0.07 *p* > 0.05	3.3	2.32 ± 0.08 *p* > 0.05	3.4	2.16 ± 0.08 *p* > 0.05	3.7	2.26 ± 0.10	4.4
Orange	3.37 ± 0.15 p > 0.05	4.4	3.60 ± 0.10 *p* > 0.05	2.8	3.50 ± 0.20 *p* > 0.05	5.7	3.40 ± 0.11	3.2

^2^*N.d.* not detected; Student’s test (*p*) was performed for values obtained by the biosensor approach compared to the reference one [22]

Table S2 presents the main analytical parameters of the bioelectrodes modified with Arg-sensitive enzymes and nCu.

In summary, the constructed novel ABSs based on Arg-selective enzymes have some advantages over mono and bi-enzymes as well as over the cell-based ABSs previously constructed by us using the same enzymes (Table 1).

Thus, the developed ABSs are highly sensitive and are low-cost in usage due to application in the bioselective layer of enzymes from microbial or forest fungus sources. The high sensitivity of the constructed ABSs will be promising in medical diagnostics to analyze the Arg in serum for diagnostics and cancer treatment where the expected concentration of Arg is not higher than 110 µM [5, 11].

The comparison of the main validation parameters, including linearity, regression equation, and limit of detection (LOD) for the developed bioelectrodes (Tables S1–S2) with the ABSs previously described in the literature (Table 1), demonstrated that the currently constructed ABSs possess significantly higher sensitivities, lower LODs, and broader linear ranges.

The ABSs for Arg determination, developed in the current work, reveal high sensitivities, do not require the combination of a cascade of enzymes, and have fast responses to Arg. Additionally, a great advantage of these ABSs is the simplicity and economy of the synthesis of ammonium-selective NPs if compared to the known analogues.

### Analytical properties of the constructed bioelectrodes

To test the selectivity, the developed bioelectrodes with the highest sensitivities were evaluated by analyzing responses on some compounds usually present in natural juices. The selectivity was estimated in relative units (%), and the maximal amperometric response upon injecting1 mM Arg was taken as 100% for all electrodes (Fig. S3). As shown in Fig. S3a–c, all of the constructed biosensors were highly specific to Arg, while the amperometric signal to other compounds was less than 5%, including L-Lys, L-Trp, L-Phe, and L-Pro. To test the stability of the proposed biosensors, the amperometric responses to 0.5 mM Arg in 50 mM PB, pH 7.5, or 50 mM TB, pH 8 (for ARG/urease/NPs/GCE), were measured during 14 days. The current response of the ArgO/nCdCu/GCE was shown to decrease by 30% for 5 days, while the half-life of this sensor was about 6 days (Fig. S3a). The half-life of ARG/urease/nCdCu/GCE and ADI/nCdCu/GCE was shown to be 6 and 7 days, respectively (Fig. S3b–c). The storage stability of the designed bioelectrodes is not good enough for their commercial introduction to the market, taking into account the possibly long delivery time when the sensors can lose their usability. However, the procedures presented here can be used for disposable sensors prepared and used in the same laboratories. Not all the elements of such sensors are disposable. Glassy carbon electrodes are renewable by polishing and ready for subsequent modification with nanomaterials, which can be stored intact for a long time, like lyophilized enzymes. To the best of our knowledge, neither voltammetric nor amperometric arginine sensors are available commercially. The obtained results suggest that the constructed biosensors provide good selectivity for Arg assay in juices. This makes the proposed sensors good candidates for wide applications by their further development towards improved stability.

The reproducibility of the constructed biosensors was also investigated by analyzing 0.5 mM Arg for 9 times during one day (*n* = 9). The relative standard deviation (RSD) is shown to be less than 3.5% for all biosensors, which is a permissible error for the analysis of real samples.

The responses of the independently prepared nanostructured bioelectrodes were also tested for 0.5 mM Arg using amperometry to estimate the operational stability (repeatability). The obtained results demonstrates that the constructed bioelectrodes reveal satisfactory operation stability (Table S1–S2) which is connected with impact nCu or nCdCu on activity of enzymes. Thus, to demonstrate the practical application of the biosensors, Arg content was further tested in commercial juices.

### Detection of Arg content in fruit juices

Arg is usually present in juices and wines [2, 3, 12, 54]. To validate the created ABSs, they were used to analyze the Arg content in juices in comparison with the reference arginase-based enzymatic-chemical method proposed by us earlier [22]. The results of this study are presented in Table 2. There is no significant difference between Arg content estimated using three different ABSs and the reference method (Table 2). It was demonstrated that Arg content estimated by each ABS and the reference method are in good agreement (*p* > 0.05) with strong (*R* = 0.993–0.999) correlations (Fig. S4).

The obtained results prove the accuracy of the biosensor approaches for Arg assay (differences are less 10.0%) and can be used for control of food quality.

## Conclusion

Detecting Arg in fresh and fermented foods is essential for the quality control of food additives and beverages including wine or juice. In the current paper, novel amperometric biosensors based on Arg selective enzymes—arginine oxidase, arginine deiminase—and arginase/urease in combination with redox-active ammonia nanochelators were constructed. The nCu and nCdCu which have the highest electrocatalytic activity toward ammonium were used to construct ABSs. Three types of the sensing elements contained different Arg-sensitive enzymes; nCdCu or nCu as NH_4_^+^-chemosensors revealed the good sensitivities, broad linear ranges, and satisfactory storage stabilities. The constructed bioelectrodes were successfully utilized for the amperometric monitoring of Arg in fruit juices. High correlations between Arg content determined by the developed biosensors and the enzymatic reference method were observed (*R* = 0.993–0.999). The fabricated NPs-based ABSs can be promising both for routine quantification of Arg by winemakers to control potential EC levels during wine production and for application in clinical diagnostics.

### Supplementary Information

Below is the link to the electronic supplementary material.

Supplementary file1 (DOCX 1384 KB)

## Data Availability

The authors declare that the data supporting the findings of this study are available within the paper and its Supplementary Information files.
