# Correction: Ammonium nanochelators in conjunction with arginine-specific enzymes in amperometric biosensors for arginine assay

**DOI:** 10.1007/s00604-024-06319-y

**Published:** 2024-04-11

**Authors:** Nataliya Stasyuk, Galina Gayda, Wojciech Nogala, Marcin Holdynski, Olha Demkiv, Lyubov Fayura, Andriy Sibirny, Mykhailo Gonchar

**Affiliations:** 1 Institute of Cell Biology, National Academy of Sciences of Ukraine, Lviv, 79005 Ukraine; 2 Institute of Physical Chemistry, Polish Academy of Sciences, Kasprzaka 44/52, 01-224 Warsaw, Poland; 3 Department of Biotechnology and Microbiology, Rzeszow University, 35-601 Rzeszow, Poland

**Correction: Microchimica Acta (2023) 191:47**

10.1007/s00604-023-06114-1

The article “Ammonium nanochelators in conjunction with arginine-specific enzymes in amperometric biosensors for arginine assay”, written by “Nataliya Stasyuk, Galina Gayda, Wojciech Nogala, Marcin Holdynski, Olha Demkiv, Lyubov Fayura, Andriy Sibirny, Mykhailo Gonchar”, was originally published Online First without Open Access. After publication in volume 191, issue 1, article 47, the author decided to opt for Open Choice and to make the article an Open Access publication. Therefore, the copyright of the article has been changed to “The Author(s) 2024” and the article is forthwith distributed under the terms of the Creative Commons Attribution 4.0 International License, which permits use, sharing, adaptation, distribution and reproduction in any medium or format, as long as you give appropriate credit to the original author(s) and the source, provide a link to the Creative Commons licence, and indicate if changes were made. The images or other third-party material in this article are included in the article’s Creative Commons licence, unless indicated otherwise in a credit line to the material. If material is not included in the article’s Creative Commons licence and your intended use is not permitted by statutory regulation or exceeds the permitted use, you will need to obtain permission directly from the copyright holder. To view a copy of this licence, visit http://creativecommons.org/licenses/by/4.0/.

The original article has been corrected.

