# Supplementary material for: Ammonium nanochelators in conjunction with arginine-specific enzymes in amperometric biosensors for arginine assay
Source: Mikrochim Acta. 2023 Dec 22;191(1):47. doi: 10.1007/s00604-023-06114-1 (PMC10987348; doi:10.1007/s00604-023-06114-1)
Supplement: Supplementary file 1 — Supplementary file1 (DOCX 1384 KB) [file 604_2023_6114_MOESM1_ESM.docx]

**Supplementary information**

**Ammonium nanochelators in conjunction with arginine- sPECIFIC enzymes in amperometric biosensors for arginine assay**

**Nataliya Stasyuk^a*^, Galina Gayda^a^, Wojciech Nogala^b*^, Marcin Holdynski^b^,**

**Olha Demkiv^a^, Lyubov Fayura^a^, Andriy Sibirny^a,c^, Mykhailo Gonchar^a^**

*^a^Institute of Cell Biology, National Academy of Sciences of Ukraine, 79005 Lviv, Ukraine;*

*^b^Institute of Physical Chemistry, Polish Academy of Sciences, 01-224 Warsaw, Poland;*

*^c^Department of Biotechnology and Microbiology, Rzeszow University, Cwiklinskiej 2, 35-601 Rzeszow, Poland*

*Corresponding authors:

stasuk_natalia@ukr.net (Nataliya Stasyuk);

wnogala@ichf.edu.pl (Wojciech Nogala)

mykhailo1952@gmail.com (Mykhailo Gonchar)

|  |  |
| --- | --- |
| (*a*) | (*b*) |
|  |  |
| (*c*) | (*d*) |
|  |  |
| (*e*) | (*f)* |
|  | |
| (*g*) | |

Figure S1. CVs (*a - f*) under injected NH_4_Cl up to 0 (1), 0.04 (2), 0.08 (3) mM, at the scan rate 10 mV·s^-1^ for NPs/GCEs electrodes: nCdCu (*a*), nCu (*b*), nCd (*c*), nCuCeAu (*d*), nCuCe (*e*) and nZnCu (*f*); the amperometric signals of different NPs/GCEs to the added ammonium chloride. The highest signal at -150 mV was chosen as 100% (*g*).

|  |  |
| --- | --- |
| _(_*_a_*_)_ | _(_*_b_*_)_ |
|  |  |
| (*c*) | (*d*) |

Figure S2. Amperometric responses of nCdCu/GCE (*a*–*c*) and nCu/GCE (*d*–*f*) on the addition of NH_4_Cl (*b, e*) and the calibration curves (*c, f*) at working potentials –0.15 V (*b,* *c*) and –0.3 V (*e,* *f*).

|  |  |  |
| --- | --- | --- |
| (*a*) | (*b*) | (*c*) |
|  |  |  |
| (*d*) | (*e*) | (*f*) |

Figure S3. Characteristics of the developed biosensors: ArgO/nCdCu/GCE (*a*, *d*), ADI/nCdCu/GCE (*b*, *e*) and ARG-urease/nCdCu/GCE (*c*, *f*): selectivity (*a*–*c*) and storage stability (*d* – *f*). Response to 1 mM analytes in 50 mM PB, pH 7.5. The highest current response was chosen as 100% in all experiments. Conditions: 22^∘^C, applied potentials: –200 mV (*a*, *e*), –150 mV (c, f).

|  |  |  |
| --- | --- | --- |
| (*a*) | (*b*) | (*c*) |

Figure S4. Correlation between the results of Arg assay in juices by the reference method and by the developed biosensors: ArgO/nCdCu/GCE (*a*), ADI/nCdCu/GCE (*b*) and ARG/urease/nCdCu/GCE (*c*). The tested juices: apple (1), raspberry (2), orange (3).

Table S1. Performances of the fabricated Arg-sensitive biosensors

| Analytical characteristics | ABSs | | |
| --- | --- | --- | --- |
|  | ArgO/nCdCu/GCE | ARG/urease/nCdCu/GCE | ADI/nCdCu/GCE |
| Working potential, mV | –150 | –150 | –200 |
| Sensitivity, A·M^-1^·m^-2^ | 1700 ± 80 | 4500 ± 170 | 1650 ± 75 |
| Linear range, µM | 30 – 160 | 2 – 80 | 12 – 200 |
| LOD, µM | 5.5 | 0.6 | 3.6 |
| LOQ, µM | 15 | 1.7 | 9.5 |
| K_M_^app^, mM | 0.27 ± 0.01 | 0.14 ± 0.004 | 0.70 ± 0.02 |
| I_max_, nA | 4510 ± 145 | 6650 ± 60 | 10460 ± 160 |
| Response time, s | 3 | 6 | 3 |
| Operational stability  (repeatability) | After the five use, 75.2% | After the five use, 88.0% | After the five use, 87.1% |

Table S2. Performances of the constructed enzyme/nCu/GCEs.

| Analytical characteristics | Biosensor | | |
| --- | --- | --- | --- |
|  | ArgO/nCu/GCE | ARG/urease/nCu/GCE | ADI/nCu/GCE |
| Working potential, mV | –300 | –300 | –300 |
| Sensitivity, A·M^-1^·m^-2^ | 2300 ± 15 | 780 ± 55 | 505 ± 3 |
| Linear range, µM | 2.5 – 18 | 6 – 50 | 6 – 130 |
| LOD, µM | 0.7 | 1.75 | 1.80 |
| LOQ, µM | 2.2 | 5.5 | 5.3 |
| K_M_^app^, mM | 0.04 ± 0.006 | 0.09 ± 0.005 | 0.15 ± 0.01 |
| I_max_, nA | 740 ± 40 | 702 ± 15 | 1130 ± 24 |
| Response time, s | 3 | 6 | 3 |
| Operational stability  (repeatability) | After the five use, 82.05% | After the five use, 80.05% | After the five use, 93.11% |
